# Preparation of Polyetherimide Nanoparticles by a Droplet Evaporation-Assisted Thermally Induced Phase-Separation Method

**DOI:** 10.3390/polym13101548

**Published:** 2021-05-12

**Authors:** Peng Zhu, Huapeng Zhang, Hongwei Lu

**Affiliations:** College of Textile Science and Engineering (International Institute of Silk), Zhejiang Sci-Tech University, Hangzhou 310018, China; roczhp@163.com (H.Z.); 2018327100068@mails.zstu.edu.cn (H.L.)

**Keywords:** droplet evaporation, polyetherimide, polymer nanoparticle, thermally induced phase separation, evaporation kinetic, coffee ring effect

## Abstract

The droplet evaporation effect on the preparation of polyetherimide (PEI) nanoparticles by thermally induced phase separation (TIPS) was studied. PEI nanoparticles were prepared in two routes. In route I, the droplet evaporation process was carried out after TIPS. In route II, the droplet evaporation and TIPS processes were carried out simultaneously. The surface tension and shape parameters of samples were measured via a drop shape analyzer. The Z-average particle diameter of PEI nanoparticles in the PEI/dimethyl sulfoxide solution (DMSO) suspension at different time points was tested by dynamic light scattering, the data from which was used to determine the TIPS time of the PEI/DMSO solution. The natural properties of the products from both routes were studied by optical microscope, scanning electron microscope and transmission electron microscope. The results show that PEI nanoparticles prepared from route II are much smaller and more uniform than that prepared from route I. Circulation flows in the droplet evaporation were indirectly proved to suppress the growth of particles. At 30 °C, PEI solid nanoparticles with 193 nm average particle size, good uniformity, good separation and good roundness were obtained. Route I is less sensitive to temperature than route II. Samples in route I were still the accumulations of micro and nanoparticles until 40 °C instead of 30 °C in route II, although the particle size distribution was not uniform. In addition, a film structure would appear instead of particles when the evaporation temperature exceeds a certain value in both routes. This work will contribute to the preparation of polymer nanoparticles with small and uniform particle size by TIPS process from preformed polymers.

## 1. Introduction

Polymer nanoparticles have attracted much attention because of their potential applications in diverse fields such as drug delivery, biosensors, photo-electrical device, coating materials, and toughening fillers [1,2,3]. Polymer nanoparticles can be directly synthesized by the polymerization of monomers using various polymerization techniques such micro-emulsion, mini-emulsion, surfactant-free emulsion and interfacial polymerization [4]. However, polymerization techniques are generally used for polymers with a simple polymerization process and in which catalysts and the excess monomers need to be removed [5].

Polymer nanoparticles can also be conveniently prepared from preformed polymers [6]. Methods such as solvent evaporation [7], nanoprecipitation [8,9], salting-out [10], dialysis [11], supercritical fluid technology [12] and thermally induced phase separation [13], can be used for the preparation of polymer nanoparticles from preformed polymers.

Thermally induced phase separation (TIPS) is related to the temperature-dependent solubility of polymers [14]. At an elevated temperature (lower than the upper critical solution temperature or higher than the lower critical solution temperature of the polymer/diluent system), the polymer can be dissolved to form a homogeneous solution, while with decreasing or increasing temperature the polymer’s solubility decreases. At a certain temperature, the polymer is no longer soluble, and phase separation is induced [15]. At a polymer concentration above the critical composition, droplets of polymer lean phase separate from the polymer rich continuous matrix phase, resulting in the formation of membrane structure. In a dilute polymer solution with a polymer concentration below the critical concentration, polymer rich phase droplets nucleate and grow from the polymer lean phase that forms the continuous matrix. The formation of spherical shape polymer particles is favored [16]. 

Therefore, TIPS can be used to prepare polymer particles in addition to filter membranes. In Hou’s research, Nylon 6 polymer microspheres were prepared from a Nylon 6/theta solution (formic acid and D.D.I. water mixture) [17]. Kim prepared nano/micro spherical poly(methyl methacrylate) particles from a poly(methyl methacrylate)/propanol solution [18]. Polypropylene particles were prepared by Matsuyama using diphenyl ether as the diluent [19].

**Figure 1:**
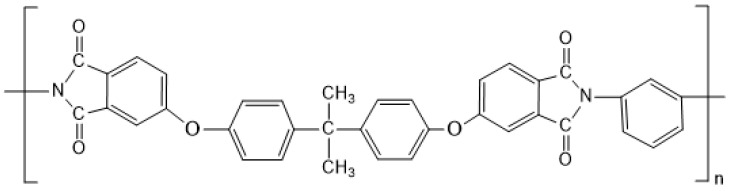
Chemical structure of PEI.

Polyetherimide (PEI) is a high-performance polymer material with good mechanical behavior, heat and radiation resistance, and hydrolytic stability [20,21]. Figure 1 shows the chemical structure of PEI, which is usually prepared of meta-substituted aromatic diamine and bisphenol-A dianhydride by high temperature polycondensation in a polar solvent [22]. Due to the complexity of the synthesis process of PEI, there is no report on the preparation of PEI micro or nanoparticles by synthetic methods. Therefore, the preparation of PEI micro or nanoparticles from preformed PEI polymer has attracted more and more attention. In Bagheri-Tar’s research, PEI nanoparticles were prepared by an electrospray technique, but the shape of the nanoparticles was irregular [23]. Giraud prepared PEI nanoparticles by emulsion/solvent evaporation, but the separation of nanoparticles was not very good, and most of the nanoparticles adhered to each other [24]. In Ding’s research, PEI microspheres with an average size of 1 μm were fabricated by water vapor-induced phase separation [25].

In our previous work, PEI nanoparticles with good roundness were successfully prepared from a PEI/DMSO by TIPS [26]. An evaporation process was used as the separation measure rather than introducing a nonsolvent. In addition, the cooling temperature, cooling time and PEI concentration were found to have a great influence on PEI nanoparticle size. However, the influence of droplet evaporation on the preparation of PEI nanoparticles was not considered. 

Research on droplet evaporation with polymer can be divided into droplet evaporation of polymer solution and droplet evaporation of polymer micro or nanoparticle suspension [27,28]. The droplet evaporation of polymer solution focuses on the influential mechanism of the evaporation process on the morphology of bulk products, which is often used in the field of ink jet printing [29]. The droplet evaporation of polymer suspension focuses on the regulation mechanism of evaporation process on the patterns of particle accumulation, which is often used in biosensors [30]. However, the effect of droplet evaporation on the evolution from polymer solution to polymer suspension, such as TIPS, has not been reported.

In this work, the effect of droplet evaporation on the preparation of PEI nanoparticles by TIPS was studied. The results show that the droplet evaporation has a great effect on the average diameter and the size distribution of PEI nanoparticles when the droplet evaporation and TIPS are carried out at the same time (DEaTIPS, route II). However, the droplet evaporation has little effect on the average diameter and the size distribution of PEI nanoparticles when the droplet evaporation is carried out after TIPS (route I). In addition, a film structure would appear instead of particles when the evaporation temperature exceeds a certain value in both routes. This DEaTIPS method will be helpful to prepare uniform polymer nanoparticles by TIPS from preformed polymers.

## 2. Materials and Methods

### 2.1. Materials

PEI (melt index 9 g/10 min [337 °C/6.6 kg]) was purchased from Sigma-Aldrich (St. Louis, MO, USA). DMSO (AR, ≥99.0%, melting point: 18.45 °C, boiling point: 189 °C) was supplied by Sinopharm Chemical Reagent Co., Ltd. (Shanghai, China). All chemicals were used as received.

### 2.2. Preparation of the PEI/DMSO Solution

A certain amount of PEI (0.2 wt.% of the DMSO solvent) was dissolved in DMSO at 90 °C to obtain the PEI/DMSO solution [26].

### 2.3. TIPS Process

The PEI/DMSO solution at 90 °C in a bottle or in the form of a droplet was placed in an environment where the temperature was lower than the upper critical solution temperature, the TIPS process appeared [19].

### 2.4. Droplet Evaporation Process

A drop of the sample solution with the volume of 3 μL was dropped on a Polytetrafluoroethylene (PTFE) thin film fixed on the hotplate by double-sided adhesive tapes. Then the droplet evaporation process was carried out at different temperature until the solvent was completely removed. The evaporation temperature was controlled from 25 °C to 50 °C by the hotplate. The setting of the evaporation temperature was based on the freezing point of the DMSO solvent and the temperature at which the PEI micro or nanoparticles disappear from the products.

### 2.5. Preparation of the PEI Nanoparticles

**Figure 2:**
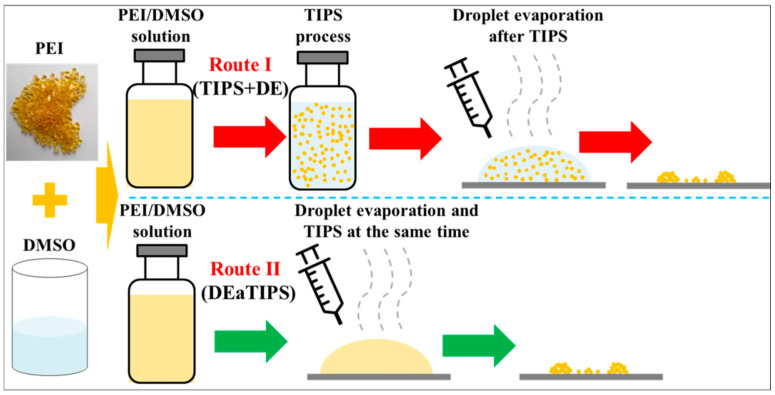
Schematic image of the two preparation routes.

To investigate the droplet evaporation effect on the preparation of PEI nanoparticles, two preparation routes were proposed. The schematic image of the two preparation routes is shown in Figure 2.

In route I, the PEI/DMSO solution was first undergoing the TIPS process by placing in an incubator at 30 °C for 20 min and 60 min, respectively. The time of TIPS process was determined according to the dynamic light scattering (DLS) test results, which would be explained in the results and discussion section. After the TIPS process, a drop of the PEI/DMSO suspension was dropped on a PTFE thin film. Then the droplet evaporation process was carried out at different temperature until the solvent was completely removed. The products are recorded as 20-TIPS + DE and 60-TIPS + DE, respectively.

In route II, a drop of the PEI/DMSO solution was dropped on a PTFE thin film. Then the TIPS and droplet evaporation processes were carried out simultaneously at different temperature until the solvent was completely removed. The products are recorded as DEaTIPS. 

In addition, the circulation flows in droplet evaporation have been confirmed by researchers [31,32], but whether it can suppress the growth of particles in the TIPS process remains to be verified by experiments. Because it is difficult to suppress the circulation flows without causing other factors to change, an experiment could be designed to indirectly confirm the influence of circulation flows on the growth of particles in the TIPS process. The verification experiment was a modification on route I. Samples were stirred at a certain speed (200 rpm) while in the TIPS process and the TIPS time was also 20 min and 60 min. The products are recorded as 20-TIPS(Stirring) + DE and 60-TIPS(Stirring) + DE, respectively. In this way, compared to 20-TIPS + DE samples and 60-TIPS + DE samples in route I, it can be concluded whether stirring has suppression effect on particle growth. Thus, the influence of circulation flows on particle growth in the DEaTIPS process could be indirectly explained.

### 2.6. Characterization

The contact angle was measured via a drop shape analyzer (Kruss DSA20, Hamburg, Germany) at room temperature. Sample droplets with volume of 3 μL were carefully dropped onto the PTFE film surface through a syringe and the contact angles were obtained by measuring five times each sample at least. The surface tension of samples was measured by the same instrument using the hanging drop method. At least five specimens were tested for each sample.

The Z-average particle diameter and polydispersity index (PDI) at different time points in the TIPS process were measured with the dynamic light scattering (DLS) method using a Malvern Zetasizer (632.8 nm, Nano ZS, Malvern Instruments, Worcestershire, UK). The TIPS process was undergoing by placing in an incubator at 30 °C.

The morphologies of the products formed after evaporation were examined using an optical microscope (OMT-1950HC, Oumit, Suzhou, China) and a scanning electron microscope (SEM, ZEISS Ultra 55, Oberkochen, Germany). The ImageJ software was used to calculate the particle size distribution in the SEM images. In addition, the natural properties were also studied by a transmission electron microscope (TEM, JEM-2100, JEOL Ltd., Tokyo, Japan).

## 3. Results and Discussion

### 3.1. Contact Angle and Surface Tension Test of Different Samples

**Figure 3:**
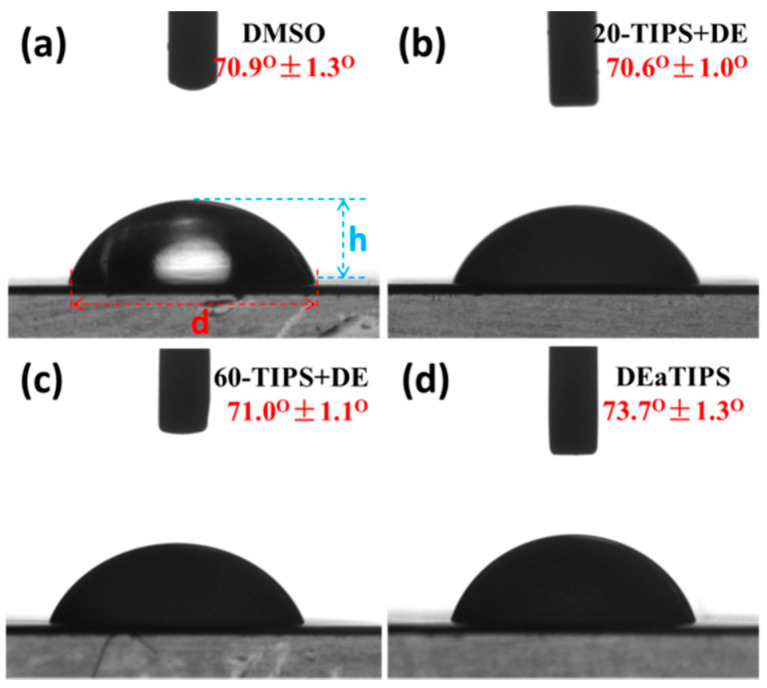
Droplet shape of (**a**) DMSO and the samples: (**b**) 20-TIPS + DE, (**c**) 60-TIPS + DE, (**d**) DEaTIPS.

Surface tension and droplet shape have been proved to have effects on droplet evaporation [31,33]. Therefore, the surface tension and shape parameters such as contact angle, droplet diameter and droplet height of the DMSO solvent and the samples were measured with a drop shape analyzer at room temperature. As show in Figure 3a–c, the contact angle of the droplet after TIPS for 20 min or more is basically the same as that of the DMSO solvent. This is because after the TIPS process, 20-TIPS + DE samples and 60-TIPS + DE samples are actually suspensions of PEI micro or nanoparticles, in which the part contacting with air and PTFE film is still the DMSO solvent. The small increase of the contact angle of DEaTIPS samples as show in Figure 3d is due to the increase of the solid-liquid interfacial tension caused by the PEI macromolecules in the PEI/DMSO suspension.

The data of surface tension and shape parameters of the DMSO solvent and the samples are list in Table 1. It can be seen that the difference of surface tension and droplet shape between the solvent and the samples are very small. It can be considered that there is no difference in the surface tension and droplet shape between the droplets in these two routes. On this basis, the effect of droplet evaporation on the TIPS process of the PEI/DMSO solution was studied in this work.

### 3.2. PEI Micro or Nanoparticle Preparation in Route I

#### 3.2.1. DLS Test of the PEI/DMSO Suspension

**Figure 4:**
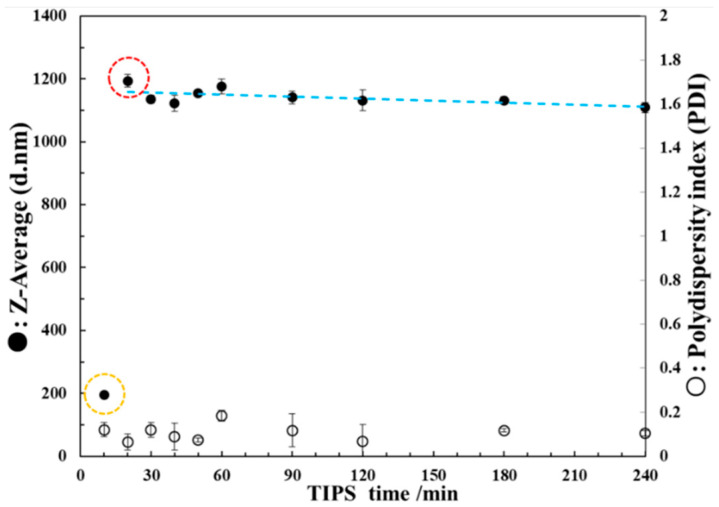
Z-average particle diameter and polydispersity index of PEI micro or nanoparticles in the PEI/DMSO suspension at different time points. The Z-average particle diameter is about 200 nm after TIPS for 10 min as shown in orange circle. The PEI/DMSO suspension was in the stage of rapid particle growth before 20 min, and reaching the maximum at 20 min as shown in red circle.

The Z-average particle diameter and polydispersity index of PEI micro or nanoparticles in the PEI/DMSO suspension at different time points were tested by DLS as shown in Figure 4. It can be seen that the Z-average particle diameter is about 200 nm after TIPS for 10 min as shown in orange circle. However, the Z-average particle diameter increases sharply to about 1200 nm after TIPS for 20 min. After that, the Z-average particle diameter tends to be stable and decreases slightly. All PDI values are very low, meaning a very narrow size distribution at each time point. The DLS data shows that the PEI/DMSO suspension was in the stage of rapid particle growth before 20 min, and reaching the maximum at 20 min as shown in red circle, meaning that the TIPS time of PEI/DMSO solution must be less than 20 min. Then the suspension entered a stable stage meaning the particles had stopped growing. The slight decrease in Z-average particle diameter in the stable stage as shown by the blue trend line should be due to the deposition of the suspension.

To investigate the droplet evaporation effect after TIPS on the preparation of PEI micro or nanoparticles, the PEI/DMSO solution must undergo a TIPS process no less than 20 min for entering the stable stage. This is the reason 20 min and 60 min were chosen as the TIPS time in route I. 

#### 3.2.2. Optical Microscope Observation of the Samples in Route I

**Figure 5:**
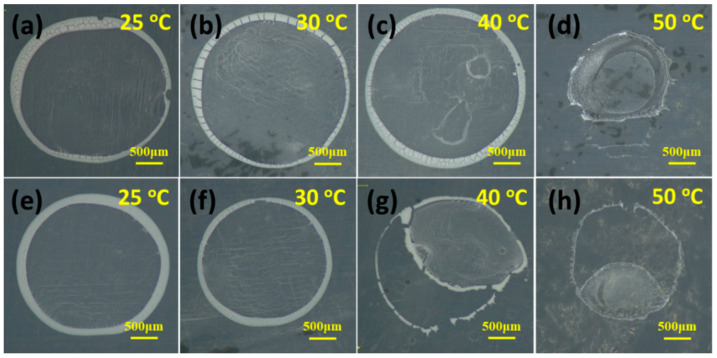
Optical microscope observation of (**a**–**d**) 20-TIPS + DE samples and (**e**–**h**) 60-TIPS + DE samples at different evaporation temperature.

Figure 5 shows the products of 20-TIPS + DE samples and 60-TIPS + DE samples after droplet evaporation at different temperature. The morphological features of 20-TIPS + DE samples and 60-TIPS + DE samples at different evaporation temperature are basically the same. When the evaporation temperature is not higher than 40 °C, the samples show the “coffee ring” pattern, which is one of the typical patterns of droplet evaporation with polymer micro or nanoparticles [30]. The samples here appear white instead of the initial brown as shown in Figure 2, which is due to the change of light refraction of 20-TIPS + DE samples and 60-TIPS + DE samples after they become micro or nanoparticles. However, when the evaporation temperature is higher than or equal to 50 °C, the samples appear a film structure.

#### 3.2.3. SEM Microscope Observation and Particle Size Distribution of the Samples in Route I

**Figure 6:**
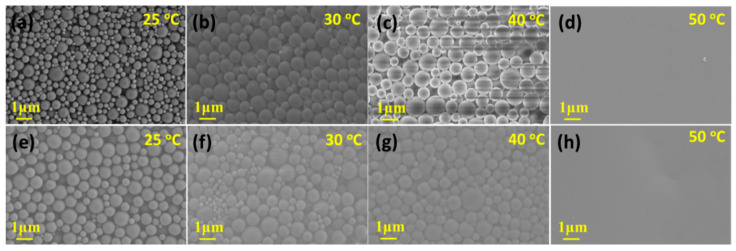
SEM observation of (**a**–**d**) 20-TIPS + DE samples and (**e**–**h**) 60-TIPS + DE samples at different evaporation temperature.

Figure 6 shows SEM images of 20-TIPS + DE samples and 60-TIPS + DE samples at different evaporation temperature. The SEM features of 20-TIPS + DE samples and 60-TIPS + DE samples at the same evaporation temperature from 25 °C to 50 °C are basically the same. When the evaporation temperature is not higher than 40 °C, the samples are the accumulations of PEI micro or nanoparticles. The particle size increases slightly with the increase of the evaporation temperature. When the evaporation temperature is higher than or equal to 50 °C, the samples both show a film structure.

**Figure 7:**
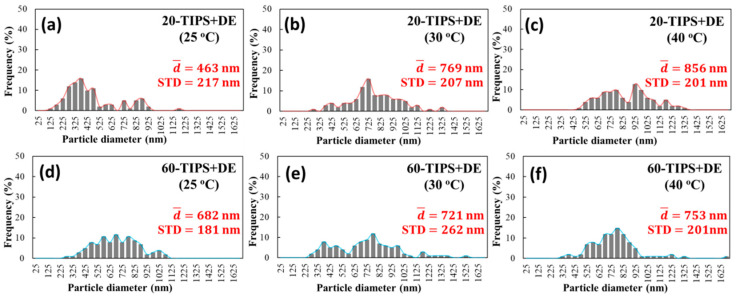
Particle size distribution of (**a**–**c**) 20-TIPS + DE samples and (**d**–**f**) 60-TIPS + DE samples at different evaporation temperature.

The particle size distribution of 20-TIPS + DE samples and 60-TIPS + DE samples was calculated by the ImageJ software as shown in Figure 7. It can be seen that the average particle diameter of both samples increases slightly with the increase of the evaporation temperature. Due to the large particle size distribution, the particle size values of 20-TIPS + DE samples and 60-TIPS + DE samples have no obvious comparative significance.

From the date above, the average diameter of PEI micro or nanoparticles after evaporation in route I was smaller than the Z-average particle diameter in the DLS test, which indicates that there must be a swelling surface layer of PEI micro or nanoparticles in the suspension; after evaporation, it shrinks, resulting in a smaller particle size. In addition, it can be seen that the particle size distribution calculated from SEM in route I is much larger than that in the DLS test. Due to the existence of the swelling surface layer, the collisions between particles are not all elastic collisions in the droplet evaporation process. There must be some inelastic collisions, and the particles entangle together to form larger particles resulting in larger particle size distribution.

### 3.3. PEI Micro or Nanoparticle Preparation in Route II

#### 3.3.1. Optical Microscope Observation of the Samples in Route II

**Figure 8:**
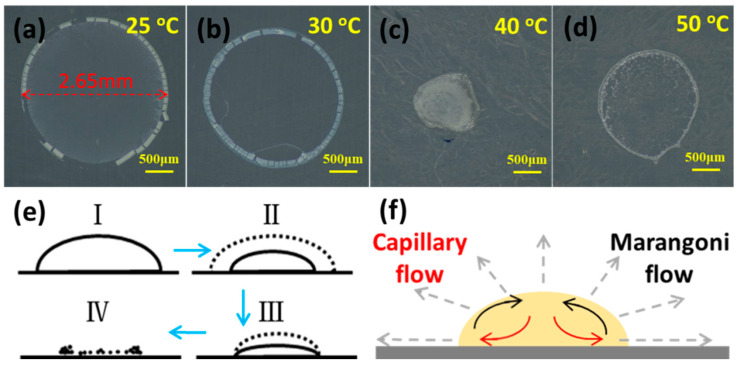
(**a**–**d**) Optical microscope observation of DEaTIPS samples at different evaporation temperature; Schematic images of (**e**) the evaporation kinetic process and (**f**) circulation flow regimes in the droplet evaporation.

Optical microscope was not only used to observe the products after droplet evaporation at different temperature, but also to infer the kinetic process during the droplet evaporation. Figure 8a–d shows the products of DEaTIPS samples after droplet evaporation at different temperature. When the evaporation temperature is 25 °C or 30 °C, DEaTIPS samples show the “coffee ring” pattern caused by the Capillary flow and interactions between the liquid and the PTFE film. DEaTIPS samples here also appear white instead of the initial brown as shown in Figure 1, which is due to the change of light refraction of DEaTIPS samples after they become micro or nanoparticles. In addition, it has a certain luster at 30 °C as shown in Figure 8b, which may be caused by the smaller and more uniform particle size. However, when the evaporation temperature is 40 °C, the DEaTIPS sample are blocky, meanwhile 20-TIPS + DE samples and 60-TIPS + DE samples are still the accumulations of micro or nanoparticles. It means that the preparation PEI micro or nanoparticles through route II is more sensitive to evaporation temperature than that through route I. When the evaporation temperature is 50 °C, the DEaTIPS sample appears a film structure, which is larger than the DEaTIPS sample at 40 °C because of the shorter evaporation time.

During the drop evaporation, two accepted theories for profile evolution are constant contact radius mode (the droplet is pinned to the surface and the height of the drop falls as the fluid evaporates) and constant contact angle mode (the radius decreases but the contact angle remains constant) [34]. In reality a combination of both usually occurs until the fluid is completely evaporated [30]. In route II, the diameter of the initial droplet is 3.5 mm, while the diameter of the final “coffee ring” pattern is 2.65 mm. Thus, it can be inferred that the droplet evaporation in route II experienced the constant contact angle evaporation first, and then the contact line was pinned and experienced the constant contact radius evaporation. Finally, the “coffee ring” pattern with diameter smaller than the initial droplet was obtained. Droplet evaporation in route I also experienced such a process. The schematic image of the evaporation kinetic process is show in Figure 8e.

A wide area of studies have worked on the effects of different circulation flows in the droplet evaporation [35,36,37]. Two important flow regimes are Capillary flow (driven by continuity) and Marangoni flow (driven by surface tension gradients) as shown in Figure 8f. The Capillary flow is assumed that evaporation occurs at the contact line of the drop and the fluid the flows radially outwards to replace the evaporated fluid. In addition, the droplet usually maintains a constant radius and the contact angle/height decrease. The deposition of particles at the contact line creates the “coffee ring” pattern. Optical microscope images (Figure 5 and Figure 8) show that the above circulation flows exist in the droplet evaporation process in both routes, which lead to the appearance of the “coffee ring” pattern.

#### 3.3.2. SEM Microscope Observation and Particle Size Distribution of the Samples in Route II

**Figure 9:**
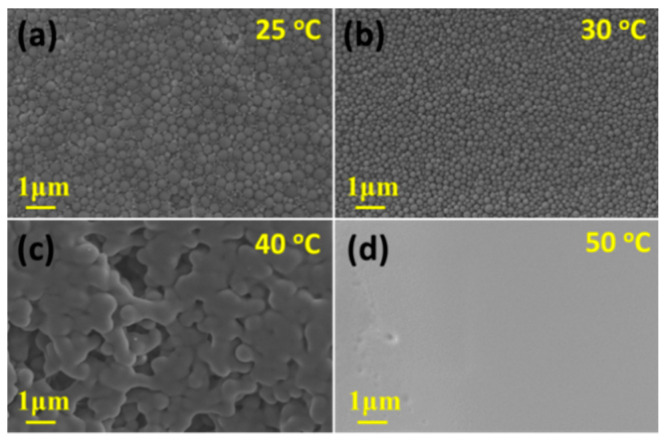
SEM images of DEaTIPS samples at different evaporation temperature: (**a**) 25 °C, (**b**) 30 °C, (**c**) 40 °C and (**d**) 50 °C.

Figure 9 shows SEM images of DEaTIPS samples prepared at different evaporation temperature. When the evaporation temperature is 25 °C or 30 °C, DEaTIPS samples are the accumulations of PEI nanoparticles. When the evaporation temperature is 25 °C, the sample shows that the large particles and small particles with obvious size difference exist at the same time (Figure 9a). There must be an internal environment that can suppress the growth of particles, but also an internal environment that is conducive to the growth of particles. It is speculated that the circulation flows are the main factor suppress the growth of particles, while the internal environment with insufficient circulation flows is conducive to the growth of particles which cannot eliminate the inelastic collision between particles.

It can be seen that a slight increase in the evaporation temperature from 25 °C to 30 °C can suppress the growth of PEI nanoparticles, forming smaller and more uniform particles as shown in Figure 9b. It can be inferred that the sufficient circulation flows caused by this slight temperature rise can eliminate the inelastic collision between particles. 

The particles become larger and stick to each other, when the evaporation temperature is 40 °C. The size of distinguishable particles is close to that in route I (Figure 6c,g), which indicates that the particle size will increase sharply with the increase of temperature from 30 °C to 40 °C, accompanying by the adhesion between particles. When the evaporation temperature is higher than or equal to 50 °C, the sample shows a film structure. With the increase of the evaporation temperature, the probability of collision between PEI nanoparticles increases, which accelerates the growth of the particles and leads to agglomerations as shown in Figure 9c, eventually leads to a film structure as shown in Figure 9d.

**Figure 10:**
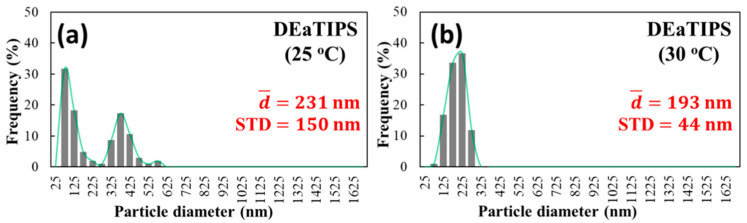
Particle size distribution of DEaTIPS samples at different evaporation temperature: (**a**) 25 °C and (**b**) 30 °C.

The ImageJ software was used to calculate the average diameter from Figure 9a,b. As shown in Figure 10, when the evaporation temperature is 25 °C and 30 °C, the average particle diameter is 231 nm and 193 nm, respectively. The appearance of the bimodal distribution in Figure 10a is due to the existence of the large particles and small particles with obvious size difference. Compared to 20-TIPS + DE samples with the evaporation temperature at 25 °C, the droplet evaporation process suppressed the growth of some particles, but at the same time, the other parts failed to suppress, resulting in obvious particle size difference. It can be considered that there are two environments in DEaTIPS samples with evaporation temperature at 25 °C, one environment suppresses the growth of particles, and the other environment does not suppress the growth of particles.

When the evaporation temperature is 30 °C, the particle size distribution is much more uniform resulting in the unimodal distribution as shown in Figure 10b. Compared to Figure 10a, it can be seen that when the temperature is slightly increased, the peak of small particle distribution and the peak of large particle distribution move towards each other and merge into a single peak. This means that the environment in DEaTIPS samples with evaporation temperature at 25 °C that suppresses the growth of particles is slightly weakened, but the environment that does not suppress the growth of particles is basically disappeared.

#### 3.3.3. TEM Microscope Observation of the Samples in Route II

**Figure 11:**
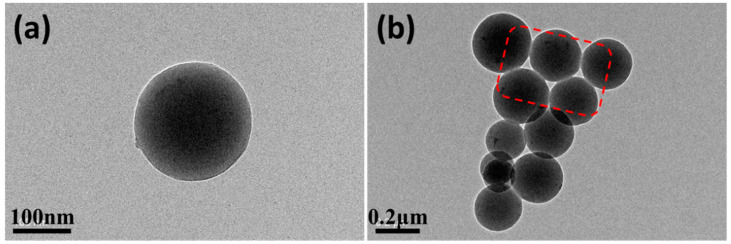
TEM images of DEaTIPS samples with the evaporation temperature at 30 °C: (**a**) a well separated nanoparticle, (**b**) a very small amount of agglomeration.

To obtain the natural properties of the PEI nanoparticles, DEaTIPS samples with the evaporation temperature at 30 °C were selected for TEM test. It can be seen that the PEI nanoparticle is a solid nanosphere with smooth surface and good roundness as shown in Figure 11a. In addition to the well separated nanoparticles, a very small amount of agglomeration can be found in TEM test (Figure 11b). As shown in the red wire frame, there are small adhesion parts between the nanoparticles, but it only exists in the surface layer, and the outline of the nanoparticles is still very clear. The adhesion between nanoparticles is due to the fact that the particles in the suspension are not completely solid particles, and the surface layer is in a swelling state, so there will be some entanglement between the particles on the surface layer, which will form a very weak adhesion when the solvent is completely evaporated. In addition, it can be separated by further grinding or other mechanical methods.

### 3.4. Effect of Stirring on Particle Growth in the TIPS Process

**Figure 12:**
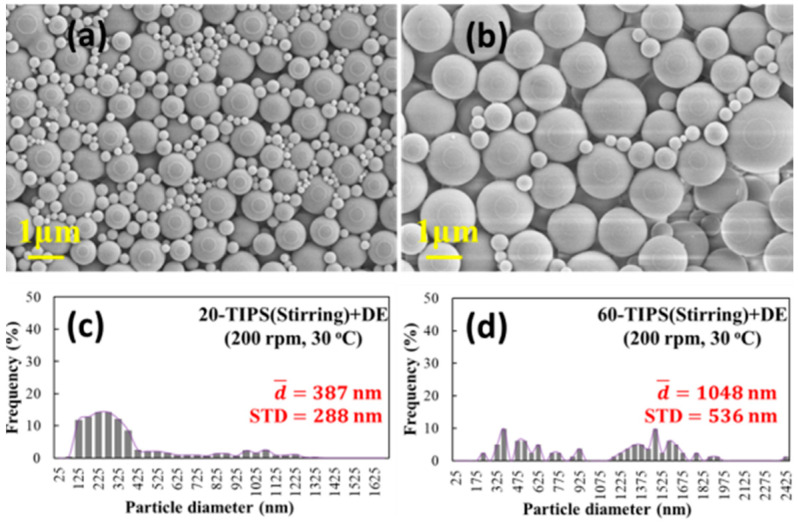
SEM observation of (**a**) 20-TIPS(Stirring) + DE samples and (**b**) 60-TIPS(Stirring) + DE samples; Particle size distribution of (**c**) 20-TIPS(Stirring) + DE samples and (**d**) 60-TIPS(Stirring) + DE samples.

To verify the effect of the circulation flows on the growth of particles in the TIPS process during droplet evaporation, the effect of stirring on particle growth in the TIPS process was studied. When the evaporation temperature is 30 °C and the TIPS time is 20 min, the particle size difference is obvious as shown in Figure 12a. Due to the large number of small particles, the distribution of 20-TIPS(Stirring) + DE samples is concentrated in the small particle area, which leads to the average particle size of 387 nm, although the standard deviation reaches 288 nm, as shown in Figure 12c.

Compared to 20-TIPS + DE samples (Figure 6b), the stirring process can obviously suppress the growth of particles, most of the particles are small particles with particle size of about 250 nm, while some particles cannot be suppressed, and large particles with particle size of about 1000 nm appear as in 20-TIPS + DE samples. However, compared to DEaTIPS samples (Figure 9b), the particles in 20-TIPS(Stirring) + DE samples are larger and not uniform, which indicates that the suppression effect of macro stirring on particle growth is not as good as that of circulation flows in the droplet evaporation.

It can be seen from Figure 12b that the particle size increases obviously when the evaporation temperature is 30 °C and the TIPS time is 60 min, and the number of small particles decreases. The average particle size increases sharply, reaching 1048 nm and the standard deviation reaches 536 nm, as shown in Figure 12d. Compared to 20-TIPS + DE samples, the particles with the stirring process here are significantly larger, which indicates that the size of PEI particles prepared from the TIPS process with a stirring process has a positive correlation with time, while that prepared from the TIPS process without stirring has no obvious relationship with time. The particle size distribution of samples at different evaporation temperature is list in Table 2.

## 4. Conclusions

In this work, DLS was used to determine the TIPS time of the PEI/DMSO solution, and then the effect of droplet evaporation on the preparation of PEI nanoparticles from the PEI/DMSO solution through two routes was studied. In route I, the droplet evaporation process was carried out after TIPS (TIPS + DE). In route II, the droplet evaporation and TIPS processes were carried out simultaneously (DEaTIPS). The DEaTIPS method was proved to prepare uniform polymer nanoparticles from preformed polymers. The following conclusions were made:

PEI nanoparticles prepared from route II are much smaller and more uniform than that prepared from route I, in which droplet evaporation process is only a means of particle separation.

1. Circulation flows in the droplet evaporation can suppress the growth of particles. At 25 °C, the growth of some particles is suppressed, while some is not; while at 30 °C, the growth of almost all particles is suppressed; PEI solid nanoparticles with 193 nm average particle size, good uniformity, good separation and good roundness were obtained.
2. A stirring process was designed to create circulation flows, which significantly suppressed the growth of particles. Therefore, it indirectly proves that the circulation flows of droplet evaporation can suppress the growth of particles in route II.
3. Route I is less sensitive to temperature than route II. Samples in route I were still the accumulations of micro and nanoparticles until 40 °C instead of 30 °C in route II, although the particle size distribution was not uniform.
4. A film structure would appear instead of particles when the evaporation temperature exceeds a certain value in both routes.

This DEaTIPS method will contribute to the preparation of polymer nanoparticles with small and uniform particle size by TIPS process from preformed polymers.

## Figures and Tables

**Table 1:** Surface tension and shape parameters of different samples.

Samples	Surface Tension (mN/m)	Contact Angle (°)	Droplet Diameter (d: mm)	Droplet Height (h: mm)
DMSO	39.1 ± 0.4	70.9 ± 1.3	3.5	1.2
20-TIPS + DE	39.3 ± 0.1	70.6 ± 1.0	3.4	1.2
60-TIPS + DE	39.0 ± 0.1	71.0 ± 1.1	3.7	1.2
DEaTIPS	39.1 ± 0.4	73.7 ± 1.3	3.5	1.2

**Table 2:** Average particle size of samples obtained by different methods.

Samples	Particle Size Distribution of Samples at Different Evaporation Temperature			
	25 °C	30 °C	40 °C	50 °C
20-TIPS + DE	463 ± 217 nm	769 ± 207 nm	856 ± 201 nm	Film structure
60-TIPS + DE	682 ± 181 nm	721 ± 262 nm	753 ± 201 nm	Film structure
DEaTIPS	231 ± 150 nm	193 ± 44 nm	Film structure	Film structure
20-TIPS(Stirring) + DE	-	387 ± 288 nm	-	-
60-TIPS(Stirring) + DE	-	1048 ± 536 nm	-	-

## References

[1] Wang Y., Fu H., Peng A., Zhao Y., Ma J., Ma Y. (2007). Distinct nanostructures from isomeric molecules of bis(iminopyrrole) benzenes: Effects of molecular structures on nanostructural morphologies. Chem. Commun..

[2] Guo B., Middha E., Liu B. (2019). Solvent Magic for Organic Particles. ACS Nano.

[3] Korokhin R.A., Solodilov V.I., Zvereva U.G., Solomatin D.V., Bamborin M.Y. (2020). Epoxy polymers modified with polyetherimide. Part II: Physicomechanical properties of modified epoxy oligomers and carbon fiber reinforced plastics based on them. Polym. Bull..

[4] Rao J.P., Geckeler K.E. (2011). Polymer nanoparticles: Preparation techniques and size-control parameters. Prog. Polym. Sci..

[5] Liu Y., Lu Y.C., Luo G.S. (2014). Modified nanoprecipitation method for polysulfone nanoparticles preparation. Soft Mater..

[6] Horn D., Rieger J. (2001). Organic nanoparticles in the aqueous phase-theory, experiment, and use. Angew. Chem. Int. Ed..

[7] Corrado I., Abdalrazeq M., Pezzella C., Di Girolamo R., Porta R., Sannia G., Giosafatto C.V.L. (2021). Design and Characterization of Poly (3-hydroxybutyrate-co-hydroxyhexanoate) Nanoparticles and Their Grafting in Whey Protein-based Nanocomposites. Food Hydrocolloid..

[8] Fessi H., Puisieux F., Devissaguet J.P., Ammoury N., Benita S. (1989). Nanocapsule Formation by Interfacial Polymer Deposition following Solvent Displacement. Int. J. Pharm..

[9] Liu Y., Yang G., Baby T., Chen D., Weitz D.A., Zhao C.X. (2020). Stable Polymer Nanoparticles with Exceptionally High Drug Loading by Sequential Nanoprecipitation. Angew. Chem. Int. Ed..

[10] Milosavljevic V., Jelinkova P., Jimenez Jimenez A.M., Moulick A., Haddad Y., Buchtelova H., Adam V. (2017). Alternative Synthesis Route of Biocompatible Polyvinylpyrrolidone Nanoparticles and Their Effect on Pathogenic Microorganisms. Mol. Pharm..

[11] Yang Z., Foster D., Dhinojwala A. (2017). Continuous Production of Polymer Nanoparticles Using a Membrane-based Flow Cell. J. Colloid Interfaces Sci..

[12] Demirdogen R.E., Emen F.M., Ocakoglu K., Murugan P., Sudesh K., Avşare G. (2018). Green Nanotechnology for Synthesis and Characterization of Poly(3-hydroxybutyrate-co-3-hydroxyhexanoate) Nanoparticles for Sustained Bortezomib Release Using Supercritical CO_2_ Assisted Particle Formation Combined with Electrodeposition. Int. J. Biol. Macromol..

[13] Hou W.H., Lobuglio T.M. (1994). A New Technique for Preparing Monodisperse Polymer Particles. II. Phase Separation Mechanisms. J. Appl. Polym. Sci..

[14] Fan S.H., Aghajani M., Wang M.Y., Martinez J., Ding Y.F. (2020). Patterning flat-sheet Poly(vinylidene fluoride) membrane using templated thermally induced phase separation. J. Membr. Sci..

[15] Liu M., Wei Y.M., Xu Z.L., Guo R.Q., Zhao L.B. (2013). Preparation and characterization of polyethersulfone microporous membrane via thermally induced phase separation with low critical solution temperature system. J. Membr. Sci..

[16] Shahzad K., Deckers J., Boury S., Neirinck B., Kruth J.P., Vleugels J. (2012). Preparation and indirect selective laser sintering of alumina/PA microspheres. Ceram. Int..

[17] Hou W.H., Lloyd T.B. (1992). A New Technique for Preparing Monodisperse Polymer Particles. J. Appl. Polym. Sci..

[18] Kim K.J. (2005). Nano/micro Spherical Poly (Methyl Methacrylate) Particle Formation by Cooling from Polymer Solution. Powder Technol..

[19] Matsuyama H., Kuwana M., Kitamura Y. (2000). Formation of polypropylene particles via thermally induced phase separation. Polymer.

[20] Khan M.Y., Khan A., Adewole J.K., Naim M., Basha S.I., Aziz M.D. (2020). Biomass derived carboxylated carbon nanosheets blended polyetherimide membranes for enhanced CO_2_/CH_4_ Separation. J. Nat. Gas Sci. Eng..

[21] Feng Y., Zhou Y.H., Zhang T.D., Zhang C.H., Zhang Y.Q., Zhang Y., Chen Q.G., Chi Q.G. (2020). Ultrahigh Discharge Efficiency and Excellent Energy Density in Oriented Core-shell Nanofiber-polyetherimide Composites. Energy Storage Mater..

[22] Meyer G.W., Tan B., Mcgrath J.E. (1994). Solvent-resistant polyetherimide network systems via phenylethynylphthalic anhydride endcapping. High Perform. Polym..

[23] Bagheri–Tar F., Sahimi M., Tsotsis T.T. (2007). Preparation of Polyetherimide Nanoparticles by an Electrospray Technique. Ind. Eng. Chem. Res..

[24] Giraud I., Franceschi-Messant S., Perez E., Lacabanne C., Dantras E. (2013). Preparation of aqueous dispersion of thermoplastic sizing agent for carbon fiber by emulsion/solvent evaporation. Appl. Surf. Sci..

[25] Ding X., Zhang P., Shu M., Gong Y., Wang Y., Zhang X., Tian X. (2019). Water vapor induced phase separation: A simple and efficient method for fabricating polyetherimide microspheres. Mater. Res. Express.

[26] Zhu P., Zhang H.P. (2021). Polyetherimide nanoparticle preparation from a polyetherimide/dimethyl sulfoxide solution by a simplified cooling-down method. Polym. Plast. Technol. Mater..

[27] Kajiya T., Monteux C., Narita T., Lequeux F., Doi M. (2009). Contact-line recession leaving a macroscopic polymer film in the drying droplets of water-poly(N,N-dimethylacrylamide) (PDMA) solution. Langmuir.

[28] Liu W.D., Midya J., Kappl M., Butt H.J., Nikoubashman A. (2019). Segregation in drying binary colloidal droplets. ACS Nano.

[29] Kim J.H., Park S.B., Kim J.H., Zin W.C. (2011). Polymer transports inside evaporating water droplets at various substrate temperatures. J. Phys. Chem. C.

[30] Khellil S. (2013). Patterns from drying drops. Adv. Colloid Interface Sci..

[31] Kajiya T., Kobayashi W., Okuzono T., Doi M. (2009). Controlling the drying and film formation processes of polymer solution droplets with addition of small amount of surfactants. J. Phys. Chem. B.

[32] Yoshitake Y., Yasumatsu S., Kaneda M., Nakaso K., Fukai J. (2010). Structure of circulation flows in polymer solution droplets receding on flat surfaces. Langmuir.

[33] Uno K., Hayashi K., Hayashi T., Ito K., Kitano H. (1998). Particle adsorption in evaporating droplets of polymer latex dispersions on hydrophilic and hydrophobic surfaces. Colloid Polym. Sci..

[34] He X.K., Cheng J.T., Collier C.P., Srijanto B.R., Briggs D.P. (2020). Evaporation of squeezed water droplets between two parallel hydrophobic/superhydrophobic surfaces. J. Colloid Interface Sci..

[35] Deegan R.D., Bakajin O., Dupont T.F., Huber G., Witten T.A. (1997). Capillary flow as the cause of ring stains from dried liquid drops. Nature.

[36] Kaneda M., Hyakuta K., Takao Y., Ishizuka H., Fukai J. (2008). Internal flow in polymer solution droplets deposited on a lyophobic surface during a receding process. Langmuir.

[37] Askounis A., Sefiane K., Shanahan M., Shanahan M.E.R. (2015). Effect of particle geometry on triple line motion of nano-fluid drops and deposit nano-structuring. Adv. Colloid Interface Sci..

